# Anticancer Activities of Newly Synthesized Chiral Macrocyclic Heptapeptide Candidates

**DOI:** 10.3390/molecules25051253

**Published:** 2020-03-10

**Authors:** Mohamed H. Abo-Ghalia, Gaber O. Moustafa, Abd El-Galil E. Amr, Ahmed M. Naglah, Elsayed A. Elsayed, Ahmed H. Bakheit

**Affiliations:** 1 Department of Peptide Chemistry, National Research Centre, Dokki 12622, Cairo, Egypt; mhaboghalia@yahoo.com (M.H.A.-G.); gosman79@gmail.com (G.O.M.); amnaglah@gmail.com (A.M.N.); 2 Pharmaceutical Chemistry Department, Drug Exploration & Development Chair (DEDC), College of Pharmacy, King Saud University, Riyadh 11451, Saudi Arabia; 3 Applied Organic Chemistry Department, National Research Centre, Dokki 12622, Cairo, Egypt; 4 Bioproducts Research Chair, Zoology Department, Faculty of Science, King Saud University, Riyadh 11451, Saudi Arabia; eaelsayed@KSU.EDU.SA; 5 Chemistry of Natural and Microbial Products Department, National Research Centre, Dokki 12622, Cairo, Egypt; 6 Department of Pharmaceutical Chemistry, College of Pharmacy, King Saud University, PO Box 2457, Riyadh 11451, Saudi Arabia; abujazz76@gmail.com; 7 Department of Chemistry, Faculty of Science and Technology, El-Neelain University, PO Box 12702, Khartoum 11121, Sudan

**Keywords:** amino acids, 3,5-tetrapeptidopyridine, macrocyclic heptapeptides, in vitro anticancer activity

## Abstract

As important cancer therapeutic agents, macrocyclic peptides have recently drawn great attention, mainly because they are synthetically accessible and have lower toxicity towards normal cells. In the present work, we synthesized newly macrocyclic pyridoheptapeptide derivatives. The synthesized derivatives were characterized using standard chemical and spectroscopic analytical techniques, and their anticancer activities against human breast and hepatocellular cancer cells were investigated. Results showed that compounds 1a and 1b were the most effective against hepatocellular (HepG2) and breast (MCF-7) cancer cell lines, respectively.

## 1. Introduction

Cancer is the second most common causative disease threatening human life. Recently, researchers have focused their works on developing successful therapeutic drugs capable of treating different cancer cells. Most research has been focused on developing early-stage cancer-treating drugs, which have received better attention in comparison to drugs used to treat late cancer phases [1]. Besides naturally obtained preparations; i.e., plant-derived extracts and microbially produced antibiotics, chemical synthesis is still used as a traditional method for obtaining potential anticancer drugs.

Among chemically synthesized pharmaceutical drugs, macrocyclic compounds with a ring of 12 or more atoms [2] are generally favored for synthesizing potential anticancer derivatives, mainly in the chemical, biological, and medical sectors [3,4,5]. Macrocyclic derivatives include peptide- and non-peptide-derived compounds, synthesized peptides, and macrocyclic derivatives [6].

**Figure 1:**
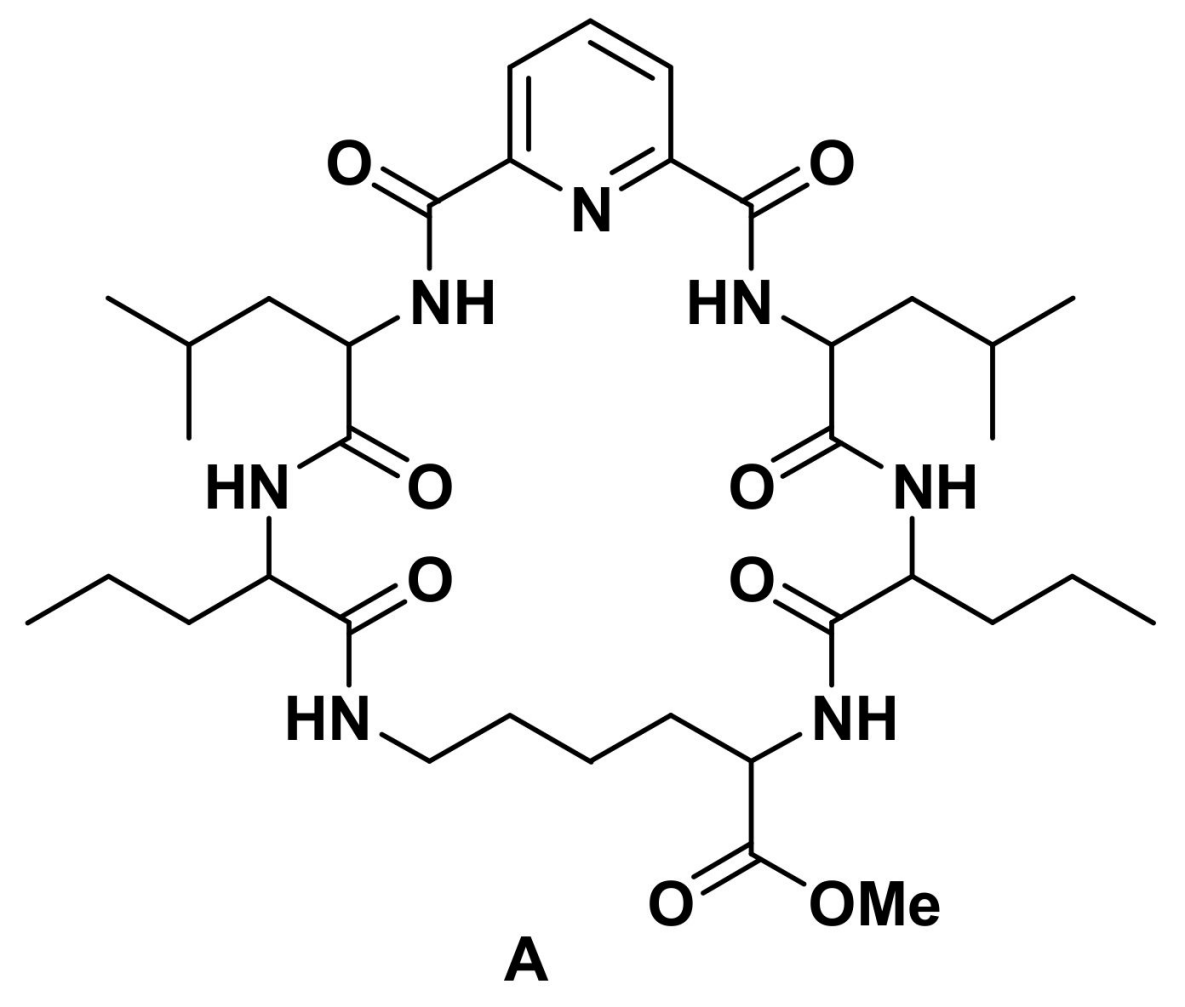
Structure of the anticancer agent.

Additionally, peptides comprise a major group of pharmaceutical drugs with potential anticancer effects [7]. Previous reports showed that chemical synthesis of peptides proved successful in obtaining new derivatives with potent antimicrobial, anti-inflammatory [8,9,10,11,12,13,14,15,16], and anticancer properties [17,18,19,20,21]. In our previous works, we were able to synthesize, chemically characterize, and biologically evaluate different bis-amino acid and peptide conjugates of dipicolinic acid [22]. Our previous work with compound (A) (Figure 1) showed potential anti-proliferative effects, mainly due to DNA intercalation, and metal sensor properties, particularly for pollutant lead (Pb2+) cations [23].

Moreover, human cells react towards toxicants; i.e., injury and infection, by developing a natural inflammatory response, which finally results in damage to the concerned tissues [24]. Inflammatory reactions include the initiation of various biological pathways such as production and secretion of pro-inflammatory mediators [25]. There has been a well-established connection between inflammatory response and cancer development. Tumor growth and development is dramatically increased by the presence of inflammatory cells; inflammatory mediators are considered a tumor microenvironment [26,27]. Recently, cancer treatments based on the application of synthesized peptides as anti-inflammatory agents have been used [28].

Based on previous investigations and our continuous work in the field of peptide synthesis [17,18,19,20,21,22,23], we have prepared different new macrocyclic heptapeptidopyridine candidates, and evaluated their anticancer potential in relation to standard used anticancer drugs.

## 2. Results and Discussion

### 2.1. Chemistry

**Scheme 1:**
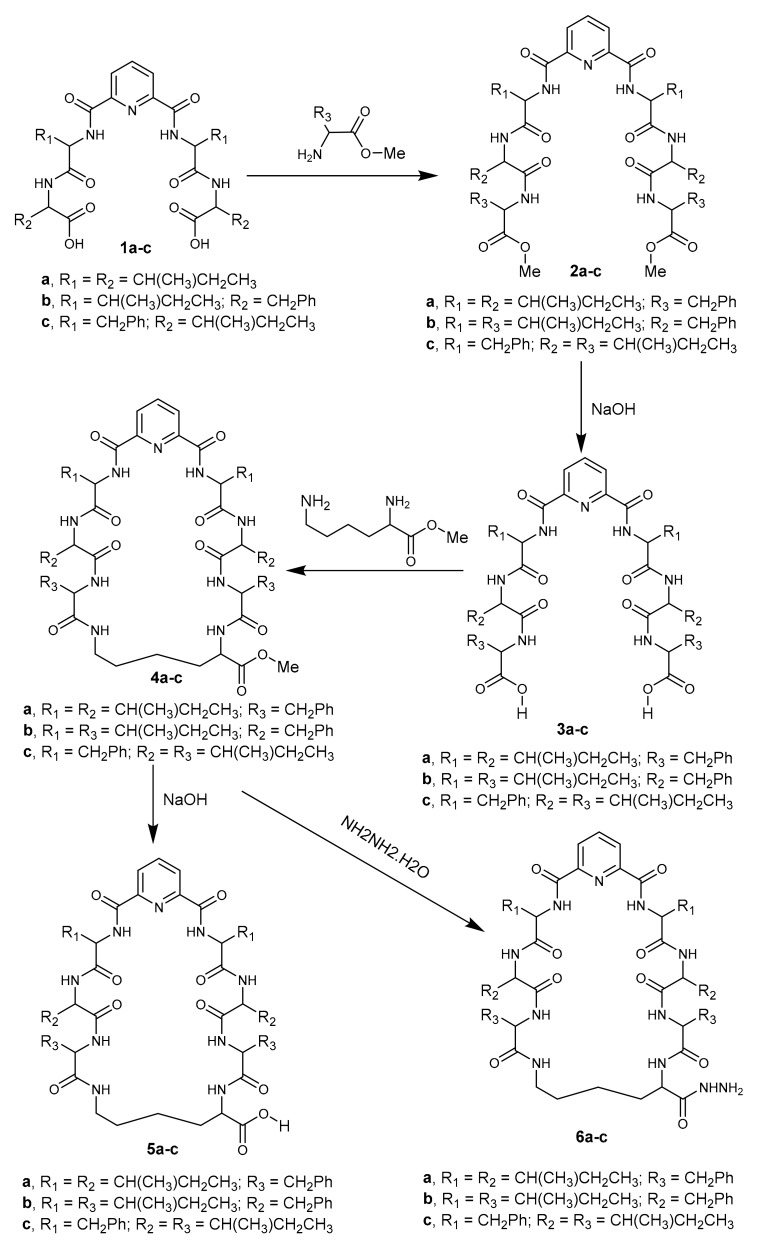
Synthetic pathway for compounds **2–6.**

In the present work we report the synthesis of newly macrocyclic pyridoheptapeptide derivatives **2–6** using *N*α-dipicolinoyl-bis[dipeptide-carboxylic acid] (**1a–c**) [29] as starting material, and they were screened as anticancer agents. Treatment of **1a-c** with *L*-amino acid methyl ester in the presence of ethyl chloroformate in dichloromethane afforded the corresponding Nα-dipicolinoyl-bis[tripeptide methyl ester] derivatives **2a–c**, respectively. The latter bis-methyl ester derivatives **2a–c** were hydrolyzed with sodium hydroxide in methanol to afford the corresponding Nα-dipicolinoyl-bis[tripeptide carboxylic acid] derivatives **3a–c**, which were cyclized with *L*-lysine methyl ester by different methods to afford the corresponding cyclo-(Nα- dipicolinoyl)-bis-[(tripeptide)-*L*-Lys- methyl ester] derivatives **4a–c**, respectively. The heptapeptide esters **3a–c** were hydrolyzed or underwent hydrazonolysis with sodium hydroxide or hydrazine hydrate in methanol to give the cyclo-(*N*α-dipicolinoyl)-bis-[(tripeptide)-*L*-Lys-carboxylic acid] derivatives **5a–c** and cyclo-(Nα-dipicolinoyl)-bis-[(tripeptide)-*L*-Lys-acid hydrazide] derivatives **6a–c**, respectively (Scheme 1).

### 2.2. Anticancer Activity

**Figure 2:**
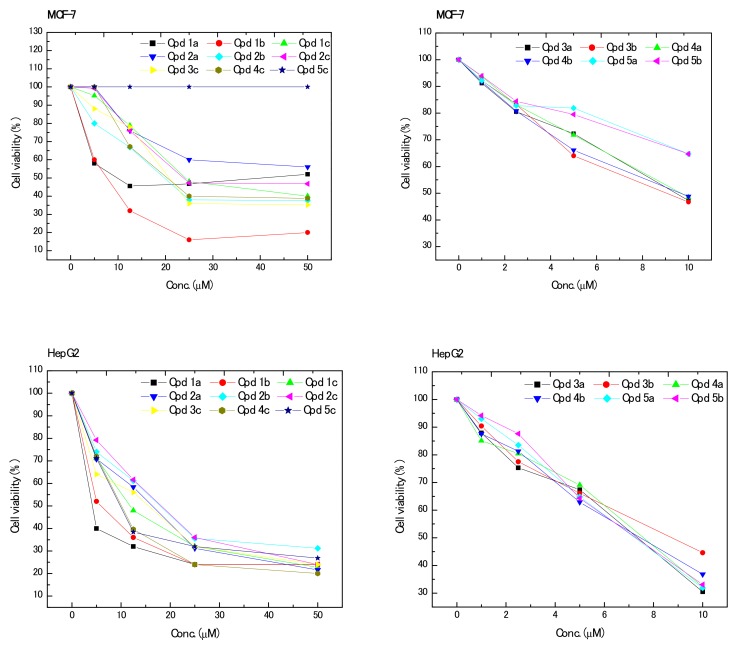
Effect of different concentrations of the prepared compounds on the viability of hepatocellular (HepG-2) and breast (MCF-7) cancer cell lines.

The newly synthesized derivatives were evaluated for their anticancer potential towards breast (MCF-7) and hepatocellular (HepG2) cancer cell lines cell lines. Compounds **6a–c** did not show any cytotoxic activity against both tested cell lines. Generally, all other derivatives, with the exception of compound 5c, showed concentration-dependent effects on both cell lines (Figure 2). Moreover, potential positive anticancer compounds showed varying effects ranging from potent to moderate effects. Increasing the applied concentration gradually increased the cytotoxic effects and correspondingly decreased cell viability. On the other hand, compound **5c** did not show any activity against MCF-7 cells, and was only active against HepG2 cells. Additionally, compounds **2a** and **5a–c** were considered practically inactive against MCF-7 cells, since they exhibited higher compound concentrations required to inhibit cell viability by 50% (IC_50_; > 100 μM) at the investigated concentration ranges. Furthermore, all compounds which exhibited anticancer activities were found to be more effective (lower IC_50_ values, Table 1) on HepG2 cells than MCF-7 cells. This can be attributed to the fact that different biological systems react differently toward same affecting compounds due to inherent morphological and membrane-structural differences among different cell lines [30,31,32]. For HepG2 cells, compounds **1a**,**b**, **3a**,**b**, **4a–c**, and **5a–c** were more potent than compounds **1c**, **2a–c**, and **3c**, which were the least effective against HepG2 cells. According to the IC_50_ values, the order of activity of the most potent compounds can be arranged as **1a** > **5b** > **5a** > **4b** > **4a** > **3a** > **1b** > **5c** > **4c** > **3b** (IC_50_: 6.62 ± 0.35, 7.09 ± 0.78, 7.12 ± 0.79, 7.17 ± 0.89, 7.31 ± 0.59, 8.06 ± 0.86, 8.73 ± 0.47, 9.57 ± 0.1.14, 9.63 ± 0.93, and 9.66 ± 0.79 µM, respectively). On the other hand, the least effective compounds showed a decreasing order of **3c** > **1c** > **2a** > **2b** > **2c** (IC_50_: 16.96 ± 1.16, 17.01 ± 0.97, 17.51 ± 0.87, 18.68 ± 0.99, and 19.32 ± 1.06 µM, respectively). On the other hand, for MCF-7 cells, compounds **1b**, **3a**,**b**, and **4a**,**b** were the most potent derivatives, where their decreasing order can be arranged as **4b** > **4a** > **3b** > **3a** > **1b** (IC_50_: 9.19 ± 0.55, 9.30 ± 0.54, 9.91 ± 0.49, 10.32 ± 0.55, and 10.90 ± 0.53 µM, respectively). Furthermore, compound **1b** showed moderate activity with an IC_50_ value of 15.33 ± 0.67 µM. Moreover, the order of the least effective compounds can be arranged as **4c** > **2b** > **3c** > **2c** > **1c** (IC_50_: 19.55 ± 1.03, 20.70 ± 0.97, 22.86 ± 0.99, 24.94 ± 1.08, and 34.89 ± 1.27 µM, respectively). On the other hand, compounds **2a** and **5a–c** were ineffective against MCF-7 cells in terms of IC_50_ values. The IC_50_ values obtained for positive controls (tamoxifen and cisplatin) were 29.34 ± 1.15 and 10.93 ± 0.96 µM for HepG2 cells, respectively, and 22.37 ± 2.41 and 8.89 ± 0.37 µM, for MCF-7 cells, respectively.

From the aforementioned results, it can be concluded that most of the prepared compounds with potent promising anticancer activities against HepG2 cells showed better activities than the positive control tamoxifen, whereas the most potent compounds against MCF-7 cells showed cytotoxic effects comparable to those of tamoxifen. Finally, it can be concluded that compounds 1a and 1b were the most potent synthesized derivatives against HepG2 and MCF-7 cells, respectively.

The structure activity relationship (SAR) was outlined in order to explain the activity of the prepared derivatives. The order of activity against cancer cell lines can be explained due to presence of free carboxylic groups, which increase the acidity, and thus increase the potential anticancer activity. Moreover, the difference in cytotoxic effects can be correlated with the substituted amino acid residues.

## 3. Materials and Methods

### 3.1. Chemistry

Melting points were determined in open glass capillary tubes with an “Electro Thermal” Digital melting point apparatus, (model: IA9100) and are uncorrected. Elemental micro-analyses results for carbon, hydrogen, and nitrogen (Microanalytical Unit, NRC) were found within the acceptable limits of the calculated values. Infrared spectra (KBr) were recorded on a Nexus 670 FTIR Nicolet, Fourier Transform infrared spectrometer. Proton nuclear magnetic resonance (^1^H NMR) spectra were run in [D_6_] DMSO on Jeol 270 MHz or 500 MHz instruments. Chemical shifts d are given in ppm. Mass spectra were run on a MAT Finnigan SSQ 7000 spectrometer, using the electron impact technique (EI). Analytical thin layer chromatography (TLC) was performed on silica gel aluminum sheets, 60 F254 (E. Merck). Specific optical rotations were measured with a A. Krawss, Optronic, P8000a polarimeter, in a 1-dm length observation tube at the indicated conditions, and according to the equation: [a]T D = 100. a = (*c l*), where: a = observed rotation angle, D = sodium line (l = 589 nm), *c* = concentration (g = 100 mL), *l* = path length in dm, and *T* = temperature (°C). The following solvent systems (by volume) were used as eluents for the development of the plates: S: chloroform-methanol-acetic acid (85: 10: 5); S_1_: S-petroleum ether (40–60 °C) (1:1); S_2_: S-petroleum ether (40–60 °C) (3: 2); S_3_: S-petroleum ether (40–60 °C) (1:2), and S_4_: butanol-water-acetic acid-pyridine (120:48:12:40). It is generally known that basic reaction media enhance racemization. However, under the reaction conditions employed in this work, especially with short reaction times and temperatures below 0 °C, only negligible racemization was observed.

#### 3.1.1. Synthesis of N^α^-dipicolinoyl-bis[tripeptide methyl ester] derivatives (**2a**–**c**)

To a cold (−15 °C) suspension of N^α^-dipicolinoyl-bis[dipeptide-carboxylic acid] (**1a**–**c**) [29] (1 mmol) and *N*-methylmorpholine (0.2 mL, 2 mmol) in dichloromethane (25 mL), ethyl chloroformate (0.2 mL, 2 mmol) was added with stirring at the same temperature (−15 °C). The reaction mixture was stirred for 20 min. Then, a cold dichloromethane solution (20 mL) of the free amino acid methyl ester of L-Phe and/or L-ILeu (2 mmol) was added, with stirring for 3 h at −15 °C and then for 12 h at room temperature. The reaction mixture was washed with water, 1 N sodium bicarbonate, and 1 N hydrochloric acid and water, and dried over anhydrous calcium chloride. The solvent was evaporated under reduced pressure; the obtained residue was triturated with dry ether/*n*-hexane mixture. The obtained solid was filtered off and crystallized from ethanol/*n*-hexane to give the corresponding bis-esters (**2a**–**c**), respectively.

*N^α^-Dipicolinoyl-bis[L-ILe-L-ILe-L-Phe-methyl ester]* (**2a**). Yield: 66%; m.p. 96–98 °C, [*α*]: -103 (c, 0.02, MeOH). IR (KBr, cm^−1^): *ν* = 3399 (NH str.), 2964 (C-H, arom.), 2872 (C-H, aliph.), 1660 (C=O, ester), 1528, 1449, 1379 (C=O amide I, II and III, respectively). 8.56–8.50 (m, 3H, Pyr-H), 8.60, 8.40, 8.36 (3s, 6H, 6NH, D_2_O exchangeable), 7.25–7.14 (m, 10H, Ar-H, L-Phe-ala), 4.68 (t, 2H, NHCH, L-Phe-ala), 4.45-4.33 (m, 4H, 4NHCH, L-Ile), 4.12 (d, 4H, 2CH_2_, L-Phe-ala), 3.60 (s, 6H, 2OCH_3_), 3.22-3.18 (m, 4H, 4NHCHCH, L-Ile), 1.20–1.12 (m, 8H, 4CH_2_, L-Ile), 0.95–0.86 (m, 24H, 8CH_3_). ^13^C-NMR (125 MHz, ppm, DMSO-d_6_): δ = 172.65 (2C, CO, ester), 172.00, 171.78 (4C, 4CO, L-Phe-ala, L-Ile), 163.74 (2C, 2CO, dicarbonyl pyridine), 147.80, 144.55, 125.80 (5C, pyr-C), 137.82, 127.90, 127.56, 125.62 (12C, Ph-C), 61.80, 58.32, 57.34 (6C, 6CHNH), 52.45 (2C, 2OCH_3_), 46.00 (2C, 2CH_2_, L-Phe-ala), 34.42, 34.10 (4C, 4CH, L-Ile), 34.12, 33.90 (4C, 4CH_2_), 14.20, 11.30 (8C, 8CH_3_, L-Ile). MS (EI, 70 eV): m/z (%) = 943 (M^+^+1, 16), 642 (M^+^, 26), 704 (64), 636 (93), 605 (85), 603 (100), 577 (80), 461 (35), 302 (45), 86 (32), 57 (39), 50 (17). Analysis for C_51_H_71_N_7_O_10_ (942.15): Calcd. C, 65.02; H, 7.60; N, 10.41. Found: C, 64.96, H, 7.54, N, 10.36.

*N^α^-Dipicolinoyl-bis[L-ILe-L-L-Phe-ILe-methyl ester]* (**2b**). Yield: 73%; m.p. 150–152 °C, [*α*]: -50 (c, 0.02, MeOH). IR (KBr, cm^−1^): *ν* = 3440 (NH, str.), 3099 (C-H, arom.), 2020 (C-H, aliph.), 1710 (C=O, ester), 1600, 1580, 1496 (C=O amide I, II and III, respectively). ^1^H-NMR (500 MHz, δ, ppm, DMSO-d_6_): δ = 8.65–8.58 (m, 3H, Pyr-H), 8.46, 8.35, 8.32 (3s, 6H, 6NH, D_2_O exchangeable), 7.35–7.20 (m, 10H, Ar-H, L-Phe-ala), 4.72 (t, 2H, NHCH, L-Phe-ala), 4.50–4.34 (m, 4H, 4NHCH, L-Ile), 4.26 (d, 4H, 2CH2, L-Phe-ala), 3.62 (s, 6H, 2OCH_3_), 3.25–3.16 (m, 4H, 4NHCHCH, L-Ile), 1.25**–**1.15 (m, 8H, 4CH_2_, L-Ile), 0.94–0.82 (m, 24H, 8CH_3_). ^13^C-NMR (125 MHz, ppm, DMSO-d_6_): δ = 173.65 (2C, CO, ester), 171.00, 169.98 (4C, 4CO, L-Phe-ala, L-Ile), 162.90 (2C, 2CO, dicarbonyl pyridine), 147.72, 144.68, 125.72 (5C, pyr-C), 137.80, 127.72, 127.50, 125.60 (12C, Ph-C), 62.00, 58.40, 57.56 (6C, 6CHNH), 52.52 (2C, 2OCH_3_), 45.68 (2C, 2CH_2_, L-Phe-ala), 34.40, 34.28 (4C, 4CH, L-Ile), 34.10, 33.92 (4C, 4CH_2_), 13.82, 11.30 (8C, 8CH_3_, L-Ile). MS (EI, 70 eV): m/z (%) = 942 (M^+^, 11), 777 (80), 593 (34), 428 (58), 340 (33), 293 (32), 207 (70), 125 (100), 90 (12), 70 (33), 57 (45), 50 (85). Analysis for C_51_H_71_N_7_O_10_ (942.15): Calcd. C, 65.02; H, 7.60; N, 10.41. Found: C, 64.94, H, 7.56, N, 10.38.

*N^α^-Dipicolinoyl-bis[L-Phe-L-ILe-L-ILe-methyl ester]* (**2c**). Yield: 70%; m.p. 94–96 °C, [*α*]: -70 (c, 0.02, MeOH). IR (KBr, cm^−1^): *ν* = 3320 (NH str.), 3066 (C-H, arom.), 2966 (C-H, aliph.), 1740 (C=O, ester), 1656, 1530, 1447 (C=O amide I, II and III, respectively). ^1^H-NMR (500 MHz, δ, ppm, DMSO-d_6_): δ = 8.70–8.64 (m, 3H, Pyr-H), 8.50, 8.40, 8.30 (3s, 6H, 6NH, D_2_O exchangeable), 7.44–7.13 (m, 10H, Ar-H, L-Phe-ala), 4.80 (t, 2H, NHCH, L-Phe-ala), 4.52–4.35 (m, 4H, 4NHCH, L-Ile), 4.15 (d, 4H, J = 8.1 Hz, 2CH2, L-Phe-ala), 3.64 (s, 6H, 2OCH_3_), 3.28-3.20 (m, 4H, 4NHCHCH, L-Ile), 1.23**–**1.12 (m, 8H, 4CH_2_, L-Ile), 0.83–0.79 (m, 24H, 8CH_3_). ^13^C-NMR (125 MHz, ppm, DMSO-d_6_): δ = 172.80 (2C, CO, ester), 171.05, 169.95 (4C, 4CO, L-Phe-ala, L-Ile), 161.75 (2C, 2CO, dicarbonyl pyridine), 147.70, 144.82, 125.70 (5C, pyr-C), 137.75, 127.84, 127.60, 125.52 (12C, Ph-C), 61.85, 58.48, 57.55 (6C, 6CHNH), 52.40 (2C, 2OCH_3_), 45.60 (2C, 2CH_2_, L-Phe-ala), 34.53, 34.32 (4C, 4CH, L-Ile), 33.90, 33.80 (4C, 4CH_2_), 13.74, 11.46 (8C, 8CH_3_, L-Ile). MS (EI, 70 eV): m/z (%) = 943 (M^+^+1, 32), 942 (M^+^, 38), 829 (33), 769 (29), 685 (66), 684 (100), 656 (53), 624 (34), 511 (34), 409 (11), 370 (89), 332 (35), 268 (15), 57 (29), 51 (72). Analysis for C_51_H_71_N_7_O_10_ (942.15): Calcd. C, 65.02; H, 7.60; N, 10.41. Found: C, 64.92, H, 7.52, N, 10.34.

#### 3.1.2. Synthesis of N^α^-dipicolinoyl-bis[tripeptide carboxylic acid]derivatives (**3a**–**c**)

To a stirred and cold methanolic solution (−15 °C, 20 mL) of the corresponding tripeptide ester (**2a**–**c**) (1 mmol), sodium hydroxide (1N, 25 mL) was gradually added. The reaction mixture was stirred for 2 h at the same temperature and then for 3 h at room temperature. The solvent was distilled off under reduced pressure, and the remaining aqueous solution was cooled and acidified with 1 N hydrochloric acid to pH = 3. The obtained solid was filtered off, washed with water, dried, and crystallized from ethanol–water to give the corresponding acids (**3a**–**c**).

*N*^α^*-Dipicolinoyl-bis[L-ILe-L-ILe-L-Phe-carboxylic acid]* (**3a**). Yield: 90 %; m.p. 137–139 °C, [*α*]: -65 (C, 0.02, MeOH). IR (KBr, cm^−1^): *ν* = 3287 (NH str), 3070 (C-H, arom), 2966 (C-H, aliph), 1646 (C=O, acid), 1531, 1452, 1386 (C=O amide I, II and III, respectively). ^1^H-NMR (500 MHz, δ, ppm, DMSO-d_6_): δ = 12.55 (s, 2H, 2OH, D_2_O exchangeable), 8.52–8.48 (m, 3H, Pyr-H), 8.20**–**8.11 (3s, 6H, 6NH, D_2_O exchangeable), 7.36-7.25 (m, 10H, Ar-H), 4.50-4.46 (t, 2H, NHCH, L-Phe-ala), 4.24 (d, 4H, 4NHCH, L-Ile), 3.26 (d, 4H, 2CH_2_, L-Phe-ala), 3.16-2.98 (m, 4H, 4NHCHCH, L-Ile), 1.28–1.08 (m, 8H, 4CH_2_, L-Ile), 0.87–0.75 (m, 24H, 8CH_3_, L-Ile). ^13^C-NMR (125 MHz, ppm, DMSO-d_6_): δ = 173.85 (2C, CO, acid), 171.65, 171.60 (4C, 4CO, L-Phe-ala, L-Ile), 167.34 (2C, 2CO, Dicarbonyl pyridine), 148.70, 140.45, 124.24 (5C, pyr-C), 137.00, 128.04, 127.30, 125.90 (12C, Ph-C), 60.90, 58.86, 58.70 (6C, 6CHNH), 42.65 (2C, 2CH_2_, L-Phe-ala), 35.50, 35.00 (4C, 4CH, L-Ile), 25.04, 24.92 (4C, 4CH_2_), 13.40, 10.90 (8C, 8CH_3_, L-Ile). MS (EI, 70 eV): m/z (%) = 915 (M^+^+1, 6), 914 (M^+^, 11), 913 (7), 833 (76), 804 (42), 776 (64), 702 (44), 641 (100), 539 (44), 461 (69), 401(27), 302 (70), 86 (39), 57 (20), 50 (5). Analysis for C_49_H_67_N_7_O_10_ (914.1): Calcd. C, 64.38; H, 7.39; N, 10.73. Found: C, 64.37, H, 7.37, N, 10.70.

*N^α^-Dipicolinoyl-bis[L-ILe-L-Phe-L-ILe-carboxylic acid]* (**3b**). Yield: 82%; m.p. 222–224 °C, [*α*]: -77 (c, 0.02, MeOH). IR (KBr, cm^−1^): *ν* = 3402 (NH str), 3073 (C-H, arom), 2966 (C-H, aliph), 1652 (C=O, acid), 1530, 1453, 1387 (C=O amide I, II and III, respectively). ^1^H-NMR (500 MHz, δ, ppm, DMSO-d_6_): δ = 12.60 (s, 2H, 2OH, D_2_O exchangeable), 8.50-8.42 (m, 3H, Pyr-H), 8.24-8.15 (3s, 6H, 6NH, D_2_O exchangeable), 7.22–7.12 (m, 10H, Ar-H), 4.70–4.62 (t, 2H, NHCH, L-Phe-ala), 4.36 (d, 4H, 4NHCH, L-Ile), 3.30 (d, 4H, 2CH_2_, L-Phe-ala), 3.06-3.00 (m, 4H, 4NHCHCH, L-Ile), 1.42–1.24 (m, 8H, 4CH_2_, L-Ile), 0.86**–**0.78 (m, 24H, 8CH_3_, L-Ile). ^13^C-NMR (125 MHz, ppm, DMSO-d_6_): δ = 174.00 (2C, CO, acid), 171.650, 171.58 (4C, 4CO, L-Phe-ala, L-Ile), 166.92 (2C, 2CO, dicarbonyl pyridine), 148.85, 140.64, 124.60 (5C, pyr-C), 137.15, 128.16, 127.24, 125.70 (12C, Ph-C), 60.82, 58.84, 58.75 (6C, 6CHNH), 42.68 (2C, 2CH_2_, L-Phe-ala), 35.34, 35.06 (4C, 4CH, L-Ile), 25.10, 24.95 (4C, 4CH_2_), 13.35, 10.76 (8C, 8CH_3_, L-Ile). MS (EI, 70 eV): m/z (%) = 915 (M^+^+1, 8), 914 (M^+^, 22), 833 (M^+^, 52), 760 (31), 637 (69), 590 (100), 477 (66), 430 (44), 400 (20), 302 (70), 69 (64), 57 (25), 51 (12). Analysis for C_49_H_67_N_7_O_10_ (914.10): Calcd. C, 64.38; H, 7.39; N, 10.73. Found: C, 64.37, H, 7.37, N, 10.69.

*N^α^-Dipicolinoyl-bis[L-Phe-L-ILe-L-ILe-carboxylic acid]* (**3c**). Yield: 66%; m.p. 138–140 °C, [*α*]: -96 (c, 0.02, MeOH). IR (KBr, cm^−1^): *ν* = 3326 (NH str), 3072 (C-H, arom), 2966 (C-H, aliph), 1721 (C=O, ester), 1655, 1529, 1452 (C=O amide I, II and III, respectively). ^1^H-NMR (500 MHz, δ, ppm, DMSO-d_6_): δ = 12.54 (s, 2H, 2OH, D_2_O exchangeable), 8.62–8.52 (m, 3H, Pyr-H), 8.10–8.09 (3s, 6H, 6NH, D_2_O exchangeable), 7.32–7.22 (m, 10H, Ar-H), 4.86-4.80 (t, 2H, NHCH, L-Phe-ala), 4.40–4.18 (d, 4H, 4NHCH, L-Ile), 3.31-3.20 (d, 4H, 2CH_2_, L-Phe-ala), 3.12–3.05 (m, 4H, 4NHCHCH, L-Ile), 1.34-1.02 (m, 8H, 4CH_2_, L-Ile), 0.92–0.77 (m, 24H, 8CH_3_, L-Ile). ^13^C-NMR (125 MHz, ppm, DMSO-d_6_): δ = 173.58 (2C, CO, acid), 171.72, 171.60 (4C, 4CO, L-Phe-ala, L-Ile), 167.05 (2C, 2CO, dicarbonyl pyridine), 148.68, 140.60, 124.45 (5C, pyr-C), 137.25, 128.10, 127.36, 125.75 (12C, Ph-C), 60.68, 58.80, 58.65 (6C, 6CHNH), 42.65 (2C, 2CH_2_, L-Phe-ala), 35.45, 35.36 (4C, 4CH, L-Ile), 25.16, 24.90 (4C, 4CH_2_), 13.32, 10.92 (8C, 8CH_3_, L-Ile). MS (EI, 70 eV): m/z (%) = 914 (M^+^, 5), 913 (2), 782 (6), 641 (2), 551 (8), 396 (9), 305 (8), 234 (9), 87 (10), 59 (100), 57 (12), 50 (4). Analysis for C_49_H_67_N_7_O_10_ (914.10): Calcd. C, 64.38; H, 7.39; N, 10.73. Found: C, 64.30, H, 7.30, N, 10.70.

#### 3.1.3. Synthesis of cyclo-(N^α^-dipicolinoyl)-bis-[tripeptide]-*L*-Lys-OMe (cyclic heptapeptide methyl esters) (**4a**–**c**)

Ethyl chloroformate (0:2 mL, 2 mmol) was added to a stirred and cold (−15 °C) dichloromethane solution (20 mL) of the corresponding *N*^α^-dipicolinoyl-*bis*[tripeptide] (**3a**–**c**) (1 mmol), containing *N*-methylmorpholine (0:2 mL, 2 mmol). The reaction mixture was stirred for additional 20 min, and then a cold (−15 °C) dichloromethane solution (20 mL) of the free L-lysine methyl ester (1 mmol) was added. Stirring was maintained for 3 h at (−15 °C), and then for 12 h at room temperature. The reaction mixture was washed with water, 1 N sodium bicarbonate, and 1 N potassium hydrogen sulfate and water, and then dried over anhydrous sodium sulfate. The solvent was evaporated under reduced pressure to dryness, and the obtained oily residue was solidified by trituration with dry ether-*n*-hexane mixture. The crude product was purified by preparative thin layer chromatography using S3 as eluent to give the corresponding cyclic heptapeptide methyl esters (**4a**–**c**).

*Cyclo-(N^α^-dipicolinoyl)-bis-[(L-ILe-L-ILe-L-Phe)-L-Lys methyl ester]* (**4a**). Yield: 82%; m.p. 86–88 °C, [*α*]: -15 (c, 0.02, MeOH). IR (KBr, cm^−1^): *ν* = 3306 (NH str), 3066 (C-H, arom), 2876 (C-H, aliph), 1741 (C=O, ester), 1651, 1529, 1450, 1381 (C=O amide I, II, III and V, respectively). 8.74–8.60 (m, 3H, Pyr-H), 8.42–8.15 (4s, 8H, 8NH, D_2_O exchangeable), 7.28–7.12 (m, 10H, Ar-H), 4.65 (t, 2H, NHCH, L-Phe-ala), 4.50-4.35 (m, 4H, 4CH, L-Ile), 4.25 (t, 1H, CH, L-Lys), 4.16 (d, 4H, 2CH_2_Ph), 3.68-3.60 (m, 2H, CH_2_, NHCH_2_, L-Lys), 3.48 (s, 3H, OCH_3_), 2,80**–**2.70 (m, 4H, 4CH, L-Ile), 2.18-1.80 (m, 6H, 3CH_2_, Lys-CH_2_), 1.25-1.12 (m, 8H, 4CH_2_, L-Ile), 0.95-0.76 (m, 24H, 8CH_3_). ^13^C-NMR (125 MHz, ppm, DMSO-d_6_): δ = 173.02 (1C, CO, ester), 172.60, 172.52, 171.98 (6C, 6CO, L-Phe-ala, L-Ile), 163.82 (2C, 2CO, dicarbonyl pyridine), 147.72, 144.68, 125.84 (5C, pyr-C), 137.75, 127.94, 127.60, 125.70 (12C, Ph-C), 59.98, 58.36, 58.24, 58.00 (7C, 7CHNH), 52.12 (1C, OCH_3_, ester), 48.68 (1C, NHCH_2_, L-Lys), 46.34, 45.98 (2C, 2CH_2_, L-Phe-ala), 37.56, 37.26 (4C, 4CH, L-Ile), 35.24, 34.70 (4C, 4CH_2_), 32.28, 29.12, 21.32 (3C, 3CH_2_, Lys), 14.30, 11.30 (8C, 8CH_3_, L-Ile). MS (EI, 70 eV): m/z (%) = 1039 (M^+^+1, 8), 1038 (M^+^, 10), 857 (10), 782 (10), 565 (24), 250 (13), 69 (40), 59 (100), 57 (78), 55 (80), 53 (9). Analysis for C_56_H_79_N_9_O_10_ (1038.28): Calcd. C, 64.78; H, 7.67; N, 12.14. Found: C, 64.77, H, 7.65, N, 12.11.

*Cyclo-(N^α^-dipicolinoyl)-bis-[(L-ILe-L-Phe-L-Ile)-L-Lys methyl ester]* (**4b**). Yield: 70%; m.p. 90**–**92 °C, [*α*]: -44 (c, 0.02, MeOH). IR (KBr, cm^−1^): *ν* = 3305 (NH str), 3065 (C-H, arom), 2965 (C-H, aliph), 1740 (C=O, ester), 1655, 1528, 1450 (C=O amide I, II and III respectively). ^1^H-NMR (500 MHz, δ, ppm, DMSO-d_6_): δ = 8.60–8.52 (m, 3H, Pyr-H), 8.35-8.18 (4s, 8H, 8NH, D_2_O exchangeable), 7.26-7.18 (m, 10H, Ar-H), 4.62 (t, 2H, NHCH, L-Phe-ala), 4.48-4.37 (m, 4H, 4NHCH, L-Ile), 4.22 (t, 1H, CH, L-Lys), 4.12 (d, 4H, 2CH_2_Ph), 3.61-3.57 (m, 2H, CH_2_, NHCH_2_, L-Lys), 3.42 (s, 3H, OCH_3_), 2.86-2.78 (m, 4H, 4CH, L-Ile), 2.00–1.76 (m, 6H, 3CH_2_, Lys-CH_2_), 1.27–1.10 (m, 8H, 4CH_2_, L-Ile), 0.99–0.71 (m, 24H, 8CH_3_, L-Ile). ^13^C-NMR (125 MHz, ppm, DMSO-d_6_): δ = 173.14 (1C, CO, ester), 172.42, 172.38, 171.84 (6C, 6CO, L-Phe-ala, L-Ile), 164.80 (2C, 2CO, dicarbonyl pyridine), 147.70, 144.64, 125.80 (5C, pyr-C), 137.70, 127.90, 127.64, 125.65 (12C, Ph-C), 60.65, 58.62, 58.34, 58.06 (7C, 7CHNH), 52.18 (1C, OCH_3_, ester), 48.79 (1C, NHCH_2_, L-Lys), 46.34 (2C, 2CH_2_, L-Phe-ala), 37.55, 37.20 (4C, 4CH, L-Ile), 35.08, 34.87 (4C, 4CH_2_), 32.45, 29.18, 21.46 (3C, 3CH_2_, Lys), 14.18, 10.96 (8C, 8CH_3_, L-Ile). MS (EI, 70 eV): m/z (%) = 1039 (M^+^+1, 3), 1038 (M^+^, 6), 924 (26), 868 (17), 650 (70), 622 (50), 590 (33), 505 (40), 477 (68), 347 (36), 330 (69), 302 (100), 293 (38), 86 (59), 69 (59), 57 (25), 50 (3). Analysis for C_56_H_79_N_9_O_10_ (1038.28): Calcd. C, 64.78; H, 7.67; N, 12.14. Found: C, 64.71, H, 7.65, N, 12.12.

*Cyclo-(N^α^-dipicolinoyl)-bis-[(L-Phe-L-ILe-L-Ile)-L-Lys methyl ester]* (**4c**). Yield: 85%; m.p. 110–112 ^o^C, [*α*]: -18 (c, 0.02, MeOH). IR (KBr, cm^−1^): *ν* = 3315 (NH str), 3065 (C-H, arom), 2964 (C-H, aliph), 1658 (C=O, ester), 1528, 1447, 1379, 1241 (C=O amide I, II, III and IV, respectively). 8.72–8.60 (m, 3H, Pyr-H), 8.45–8.14 (4s, 8H, 8NH, D_2_O exchangeable), 7.30-7.16 (m, 10H, Ar-H), 4.62 (t, 2H, NHCH, L-Phe-ala), 4.54-4.38 (m, 4H, 4CH, L-Ile), 4.256 (t, 1H, CH, L-Lys), 4.18 (d, 4H, 2CH_2_Ph), 3.66-3.58 (m, 2H, CH_2_, NHCH_2_, L-Lys), 3.46 (s, 3H, OCH_3_), 2.76–2.65 (m, 4H, 4CH, L-Ile), 2.24–1.86 (m, 6H, 3CH_2_, Lys-CH_2_), 1.24–1.18 (m, 8H, 4CH_2_, L-Ile), 0.96–0.82 (m, 24H, 8CH_3_). ^13^C-NMR (125 MHz, ppm, DMSO-d_6_): δ = 173.32 (1C, CO, ester), 172.60, 172.30, 171.72 (6C, 6CO, L-Phe-ala, L-Ile), 164.75 (2C, 2CO, dicarbonyl pyridine), 147.75, 144.65, 125.85 (5C, pyr-C), 137.72, 127.93, 127.64, 125.62 (12C, Ph-C), 61.14, 58.60, 58.30, 58.00 (7C, 7CHNH), 52.44 (1C, OCH_3_, ester), 48.85 (1C, NHCH_2_, L-Lys), 46.60 (2C, 2CH_2_, L-Phe-ala), 37.58, 37.24 (4C, 4CH, L-Ile), 35.00, 34.92 (4C, 4CH_2_), 32.44, 29.22, 21.45 (3C, 3CH_2_, Lys), 14.12, 10.95 (8C, 8CH_3_, L-Ile). MS (EI, 70 eV): m/z (%) = 1039 (M^+^+1, 4), 10.38 (M^+^, 5), 925 (9), 867 (10), 812 (19), 726 (26), 684 (84), 656 (48), 571 (56), 511 (32), 409 (20), 370 (100), 86 (23), 57 (10), 51 (2). Analysis for C_56_H_79_N_9_O_10_ (1038.28): Calcd. C, 64.78; H, 7.67; N, 12.14. Found: C, 64.73, H, 7.65, N, 12.14.

#### 3.1.4. Synthesis of cyclo-(N^α^-dipicolinoyl)-bis[tripeptide]-L-Lys-carboxylic acid (**5a**–**c**)

To a stirred and cold methanolic solution (−5 °C, 20 mL) of cyclic heptapeptide methyl ester (**4a**–**c**) (1 mmol), sodium hydroxide (1 N, 25 mL) was gradually added. The reaction mixture was stirred for 2 h at the same temperature, and then for 3 h at room temperature. The solvent was distilled off under reduced pressure, and the remaining aqueous solution was cooled and acidified with 1 N hydrochloric acid to pH = 3. The obtained solid was filtered off, washed with water, dried, and crystallized from ethanol/water to give the corresponding cyclic heptapeptide methyl acids (**5a–c**).

*Cyclo-(N^α^-dipicolinoyl)-bis-[(L-ILe-L-Ile-L-Phe)-L-Lys carboxylic acid]* (**5a**). Yield: 70 %; m.p. 132–134 °C, [*α*]: -20 (c, 0.02, MeOH). IR (KBr, cm^−1^): *ν* = 3310 (NH str), 3066 (C-H, arom), 2965 (-CH, aliph), 1652 (C=O, acid), 1529, 1452, 1383, 1227 (C=O amide I, II, III, and IV, respectively). ^1^H-NMR (500 MHz, δ, ppm, DMSO-d_6_): δ = 12.50 (s, 1H, OH, D_2_O exchangeable), 8.75-8.65 (m, 3H, Pyr-H), 8.50-8.18 (m, 8H, 8NH, D_2_O exchangeable), 7.50**–**7.21 (m, 10H, Ar-H), 4.60 (t, 2H, 2 CHCH_2_, L-Phe-ala), 4.55-4.45 (m, 4H, 4CH, L-Ile), 4.35 (t, 1H, CH, L-Lys), 4.18 (t, 4H, 2CH_2_Ph), 3.75**–**3.70 (m, 2H, NHCH_2_, L-Lys), 2.72**–**2.60 (m, 4H, 4CH, L-Ile), 2.00-1.80 (m, 6H, 3CH_2_, Lys-CH_2_), 1.27**–**1.14 (m, 8H, 4CH_2_, L-Ile), 0.93**–**0.74 (m, 24H, 8CH_3_). ^13^C-NMR (125 MHz, ppm, DMSO-d_6_): δ = 174.00 (1C, CO, Acid), 172.90, 172.80, 171.16 (6C, 6CO, L-Phe-ala, L-Ile), 168.10 (2C, 2CO, dicarbonyl pyridine), 147.60, 144.45, 125.80 (5C, pyr-C), 137.70, 128.55, 127.60, 125.78 (12C, Ph-C), 59.95, 58.32, 58.22, 58.04 (7C, 7CHNH), 48.56 (1C, NHCH_2_, L-Lys), 46.30, 45.95 (2C, 2CH_2_, L-Phe-ala), 37.55, 37.22 (4C, 4CH, L-Ile), 35.16, 34.80 (4C, 4CH_2_), 32.20, 29.12, 21.30 (3C, 3CH_2_, Lys), 14.24, 11.36 (8C, 8CH_3_, L-Ile). MS (EI, 70 eV): m/z (%) = 1025 (M^+^ + 1, 22), 1024 (M^+^, 6), 816 (26), 645 (26), 562 (36), 388 (32), 221 (31), 157 (30), 91 (83), 69 (53), 59 (58), 57 (100), 55 (98), 51 (32), 50 (15). Analysis for C_55_H_77_N_9_O_10_ (1024.25): Calcd. C, 64.49; H, 7.58; N, 12.31. Found: C, 64.40, H, 7.50, N, 12.28.

*Cyclo-(N^α^-dipicolinoyl)-bis-[(L-ILe-L-Phe-L-Ile)-L-Lys carboxylic acid]* (**5b**). Yield: 65 %; m.p. 138–140 °C, [*α*]: -45 (c, 0.02, MeOH). IR (KBr, cm^−1^): *ν* = 3312 (NH str.), 3065 (C-H, arom), 2965 (C-H, aliph), 1654 (C=O, acid), 1529, 1451, 1384 (C=O amide I, II and III respectively). ^1^H-NMR (500 MHz, δ, ppm, DMSO-d_6_): δ = 12.45 (s, 1H, OH, D_2_O exchangeable), 8.95-8.85 (m, 3H, Pyr-H), 8.42–8.12 (s, 8H, 8NH, D_2_O exchangeable), 7.45–7.30 (m, 10H, Ar-H), 4.70 (t, 2H, 2CHCH_2_, L-Phe-ala), 4.40–4.36 (m, 4H, 4CH, L-Ile), 4.25 (t, 1H, CH, L-Lys), 4.05 (t, 4H, 2CH_2_Phe), 3.70**–**3.62 (m, 2H, CH_2_, NHCH_2_, L-Lys), 2.80–2.70 (m, 4H, 4CH, L-Ile), 2.00–1.45 (m, 6H, 3CH_2_, Lys-CH_2_), 1.35–1.15 (m, 8H, 4CH_2_, L-Ile), 0.90–0.71 (m, 24H, 8CH_3_). ^13^C-NMR (125 MHz, ppm, DMSO-d_6_): δ = 173.85 (1C, CO, Acid), 173.65, 173.33, 170.35 (6C, 6CO, L-Phe-ala, L-Ile), 167.60 (2C, 2CO, dicarbonyl pyridine), 147.66, 144.18, 125.80 (5C, pyr-C), 137.90, 128.57, 127.75, 125.60 (12C, Ph-C), 60.16, 58.28, 58.18, 58.08 (7C, 7CHNH), 48.28 (1C, NHCH_2_, L-Lys), 46.26, 45.95 (2C, 2CH_2_, L-Phe-ala), 37.56, 37.28 (4C, 4CH, L-Ile), 35.00, 34.78 (4C, 4CH_2_), 32.18, 29.05, 21.08 (3C, 3CH_2_, Lys), 14.75, 11.26 (8C, 8CH_3_, L-Ile). MS (EI, 70 eV): m/z (%) = 1025 (M^+^+1, 26), 789 (19), 654 (25), 514 (22), 330 (35), 69 (52, 59 (54), 57 (100), 54 (22). Analysis for C_55_H_77_N_9_O_10_ (1024.25). Calcd. C, 64.49; H, 7.58; N, 12.31. Found: C, 64.44, H, 7.56, N, 12.30.

*Cyclo-(N^α^-dipicolinoyl)-bis-[(L-Phe-L-ILe-L-Ile)-L-Lys carboxylic acid]* (**5c**). Yield: 90%; m.p. 147–149 °C, [*α*]: -41 (c, 0.02, MeOH). IR (KBr, cm^−1^): *ν* = 3322 (NH str.), 3065 (C-H, arom), 2965 (C-H, aliph), 1600 (C=O, acid), 1527, 1449, 1383, 1231 (C=O amide I, II, III and IV, respectively). ^1^H-NMR (500 MHz, δ, ppm, DMSO-d_6_): δ = 12.65 (s, 1H, OH, D_2_O exchangeable), 8.86–8.70 (m, 3H, Pyr-H), 8.56–8.22 (m, 8H, 8NH, D_2_O exchangeable), 7.41–7.35 (m, 10H, Ar-H), 4.75 (t, 2H, 2CHCH_2_, L-Phe-ala), 4.60-4.42 (m, 4H, 4CH, L-Ile), 4.35 (t, 1H, CH, L-Lys), 3.96 (t, 4H, 2CH_2_Phe), 3.65**–**3.55 (m, 2H, CH_2_, NHCH_2_, L-Lys), 2.90–2.75 (m, 4H, 4CH, L-Ile), 2.00–1.65 (m, 6H, 3CH_2_, Lys-CH_2_), 1.30–1.10 (m, 8H, 4CH_2_, L-Ile), 0.90–0.76 (m, 24H, 8CH_3_). ^13^C-NMR (125 MHz, ppm, DMSO-d_6_): δ = 174.02 (1C, CO, Acid), 173.18, 173.06, 170.15 (6C, 6CO, L-Phe-ala, L-Ile), 167.60 (2C, 2CO, Dicarbonyl pyridine), 147.95, 144.12, 126.15 (5C, pyr-C), 137.60, 128.40, 127.80, 125.72 (12C, Ph-C), 59.85, 58.60, 58.45, 58.28 (7C, 7CHNH), 48.32 (1C, NHCH_2_, L-Lys), 46.42, 45.94 (2C, 2CH_2_, L-Phe-ala), 37.62, 37.54 (4C, 4CH, L-Ile), 35.12, 34.88 (4C, 4CH_2_), 32.25, 29.16, 21.18 (3C, 3CH_2_, Lys), 14.52, 11.26 (8C, 8CH_3_, L-Ile). MS (EI, 70 eV): m/z (%) = 1025 (M^+^+1, 2), 10.24 (M^+^, 4), 689 (2), 621 (26), 565 (12), 406 (5), 113 (4), 59 (100), 57 (21), 50 (8). Analysis for C_55_H_77_N_9_O_10_ (1024.25): Calcd. C, 64.49; H, 7.58; N, 12.31. Found: C, 64.43, H, 7.55, N, 12.29.

#### 3.1.5. Synthesis of cyclo-[(N^α^-dipicolinoyl)-bis-(tripeptide)-L-Lys-NHNH_2_] (cyclic heptapeptide hydrazides) (**6a**–**c**).

To a stirred methanolic solution (20 mL) of the corresponding cyclic pentapeptide methyl ester (**4a**–**c**) (1 mmol), anhydrous hydrazine hydrate (0:35 mL, 10 mmol) was added with refluxing for 3 h. The solvent was evaporated and the obtained residue was triturated with ether, filtered off, and crystallized from methanol/ether to afford the hydrazides (**6a**–**c**).

*Cyclo-(N^α^-dipicolinoyl)-bis-[(L-Ile-L-Ile-L-Phe)-L-Lys acid hydrazide]* (**6a**). Yield: 75%; m.p. 168–170 °C, [*α*]: -35 (c, 0.02, MeOH). IR (KBr, cm^−1^): *ν* = 3300 (NH str.), 3066 (C-H, arom.), 2965 (CH, aliph.), 1652, 1530, 1452 and 1385 (C=O amide I, II, III and IV, respectively). ^1^H-NMR (500 MHz, δ, ppm, DMSO-d_6_): δ = 9.40 (s, 1H, NH, D_2_O exchangeable), 8.80–8.60 (m, 3H, Pyr-H), 8.45–8.21 (m, 8H, 8NH, D_2_O exchangeable), 7.60–7.50 (m, 10H, Ar-H), 4.90 (t, 2H, 2 CHCH_2_, L-Phe-ala), 4.70–4.55 (m, 4H, 4CH, L-Ile), 4.35 (t, 1H, CH, L-Lys), 4.25 (s, 2H, NH_2_, D_2_O exchangeable), 4.15 (t, 4H, 2CH_2_Ph), 3.85–3.80 (m, 2H, CH_2_, L-Lys), 2.35–2.20 (m, 4H, 4CH, L-Ile), 1.60–1.40 (m, 6H, 3CH_2_, Lys-CH_2_), 1.25–1.00 (m, 8H, 4CH_2_, L-Ile), 0.90–0.75 (24H, 8CH_3_). ^13^C-NMR (125 MHz, ppm, DMSO-d_6_): δ = 173.16, 173.02, 170.16 (6C, 6CO, L-Phe-ala, L-Ile), 171.55 (1C, CO, Hydrazide), 167.72 (2C, 2CO, dicarbonyl pyridine), 147.65, 144.14, 125.82 (5C, pyr-C), 137.90, 128.30, 127.65, 125.70 (12C, Ph-C), 60.12, 58.25, 58.13, 58.00 (7C, 7CHNH), 48.10 (1C, NHCH_2_, L-Lys), 46.32, 45.92 (2C, 2CH_2_, L-Phe-ala), 37.56, 37.24 (4C, 4CH, L-Ile), 35.12, 34.68 (4C, 4CH_2_), 32.18, 29.05, 21.04 (3C, 3CH2, Lys), 14.70, 11.30 (8C, 8CH_3_, L-Ile). MS (EI, 70 eV): m/z (%) = 1038 (M^+^, 17), 1027 (30), 965 (33), 878 (30), 754 (42), 597 (34), 375 (36), 167 (36), 71 (58), 60 (58), 57 (100), 55 (84), 51 (14). Analysis for C_55_H_79_N_11_O_9_ (1038.28): Calcd. C, 63.62; H, 7.67; N, 14.84. Found: C 63.55, H 7.60, N 14.76.

*Cyclo-(N^α^-dipicolinoyl)-bis-[(L-Ile-L-Phe-L-Ile)-L-Lys acid hydrazide]* (**6b**). Yield: 70%; m.p. 182–184 °C, [*α*]: -55 (c, 0.02, MeOH). IR (KBr, cm^−1^): *ν* = 3296 (NH str.), 3064 (C-H, arom.), 2931 (C-H, aliph.), 1752, 1529, 1451, 1383 (C=O amide I, II, III, IV and V, respectively). ^1^H-NMR (500 MHz, δ, ppm, DMSO-d_6_): δ = 9.35 (s, 1H, NH, D_2_O exchangeable), 8.90–8.75 (m, 3H, Pyr-H), 8.35–8.20 (m, 8H, 8NH, D_2_O exchangeable), 7.65–7.45 (m, 10H, Ar-H), 4.85 (t, 2H, 2 CHCH_2_, L-Phe-ala), 4.72–4.58 (m, 4H, 4CH, L-Ile), 4.44 (t, 1H, CH, L-Lys), 4.30 (s, 2H, NH_2_, D_2_O exchangeable), 4.12 (t, 4H, 2CH_2_Ph), 3.80–3.70 (m, 2H, CH_2_, L-Lys), 2.50–2.40 (m, 4H, 4CH, L-Ile), 1.70–1.50 (m, 6H, 3CH_2_, Lys-CH_2_), 1.30–1.05 (m, 8H, 4CH_2_, L-Ile), 0.95-0.85 (24H, 8CH_3_). ^13^C-NMR (125 MHz, ppm, DMSO-d_6_): δ = 173.30, 173.15, 170.25 (6C, 6CO, L-Phe-ala, L-Ile), 171.60 (1C, CO, Hydrazide), 167.80 (2C, 2CO, dicarbonyl pyridine), 147.7, 144.12, 125.9 (5C, pyr-C), 137.95, 128.35, 127.70, 125.75 (12C, Ph-C), 60.10, 58.25, 58.15, 58.04 (7C, 7CHNH), 48.12 (1C, NHCH_2_, L-Lys), 46.20, 45.98 (2C, 2CH_2_, L-Phe-ala), 37.5, 37.2 (4C, 4CH, L-Ile), 35.10, 34.32 (4C, 4CH_2_), 32.15, 29.00, 21.00 (3C, 3CH_2_, Lys), 14.7, 11.25 (8C, 8CH_3_, L-Ile). MS (EI, 70 eV): m/z (%) = 1039 (M^+^+1, 14), 1038 (M^+^, 19), 918 (20), 767 (21), 737 (20), 661 (60), 590 (49), 522 (84), 429 (43), 375 (63), 347 (100), 302 (68), 69 (64), 57 (85), 55 (66), 51 (16). Analysis for C_55_H_79_N_11_O_9_ (1038.28): Calcd. C, 63.62; H, 7.67; N, 14.84. Found: C 63.50, H 7.60, N 14.74.

*Cyclo-(N^α^-dipicolinoyl)-bis-[(L-Phe-L-ILe-L-Ile)-L-Lys acid hydrazide]* (**6c**). Yield: 74 %; m.p. 175–177 °C, [*α*]: -66 (c, 0.02, MeOH). IR (KBr, cm^−1^): *ν* = 3298 (NH str.), 3064 (C-H, arom.), 2964 (C-H, aliph.), 1653, 1527, 1448, 1383, 1241 (C=O amide I, II, III, IV and V, respectively). ^1^H-NMR (500 MHz, δ, ppm, DMSO-d_6_): δ = 9.35 (s, 1H, NH, D_2_O exchangeable), 9.05–8.60 (m, 3H, Pyr-H), 8.10–8.00 (m, 8H, 8NH, D_2_O exchangeable), 7.50–7.15 (m, 10H, Ar-H), 4.86 (t, 2H, 2 CHCH_2_, L-Phe-ala), 4.50-4.40 (m, 4H, 4CH, L-Ile), 4.25 (t, 1H, CH, L-Lys), 4.05 (s, 2H, NH_2_, D_2_O exchangeable), 3.98 (t, 4H, 2CH_2_Ph), 3.70 (t, 2H, CH_2_, L-Lys), 2.80–2.65 (m, 4H, 4CH, L-Ile), 1.90–1.70 (m, 6H, 3CH_2_, Lys-CH_2_), 1.35**–**1.12 (m, 8H, 4CH_2_, L-Ile), 1.00–0.82 (24H, 8CH_3_). ^13^C-NMR (125 MHz, ppm, DMSO-d_6_): δ = 173.36, 173.16, 170.10 (6C, 6CO, L-Phe-ala, L-Ile), 171.580 (1C, CO, Hydrazide), 167.56 (2C, 2CO, dicarbonyl pyridine), 148.5, 144.0, 126.1 (5C, pyr-C), 137.78, 128.42, 127.76, 125.80 (12C, Ph-C), 60.00, 58.34, 58.18, 58.06 (7C, 7CHNH), 48.18 (1C, NHCH_2_, L-Lys), 46.35, 45.95 (2C, 2CH_2_, L-Phe-ala), 37.6, 37.3 (4C, 4CH, L-Ile), 35.16, 34.38 (4C, 4CH_2_), 32.22, 29.04, 21.08 (3C, 3CH_2_, Lys), 14.5, 11.20 (8C, 8CH_3_, L-Ile). MS (EI, 70 eV): m/z (%) = 1038 (M^+^, 8), 10.37 (12), 926 (24), 778 (22), 664 (25), 624 (43), 522 (45), 471 (26), 409 (73), 370 (100), 324 (21), 165 (12), 86 (53), 57 (69), 55 (51), 50 (10). Analysis for C_55_H_79_N_11_O_9_ (1038.28): Calcd. C 63.62; H 7.67; N 14.84. Found: C 63.54, H 7.63, N 14.75.

### 3.2. Anticancer Activity

The cytotoxic effects of the prepared compounds on MCF-7 and HepG2 cells were investigated with the help of standard MTT assay [33,34]. Cells were plated in RPMI 1640 medium in 96-well culture plates 2 × 10^4^/mL, incubated for 24 h for adherence, and then different concentrations of the prepared derivatives (0–1 μM/DMSO) were added to well plates. Plates were then further incubated for 72 h. Afterwards, 20 μL of MTT (5 mg/mL in PBS) were pipetted to the wells and incubated for another 4 h. The medium was then aspirated and 100 μL DMSO were added/well. Absorbance was read using a microplate reader at 570 nm [35]. IC_50_ values, used to compare compound toxicity with control cells, were obtained from linear regression of the dose–response curve using Origin^®^ 6.1 software (OriginLab Corporation, Northampton, MA, USA). Experiments were performed in triplicates, and tamoxifen and cisplatin were used as positive control. Data were represented as mean ± SD.

## 4. Conclusions

In summary, a series of macrocyclic derivatives bearing a pyrido-heptapeptide moiety were designed and synthesized from Nα-dipicolinoyl-bis[dipeptide-carboxylic acid]. Two human cancer cell lines (MCF-7 and HepG-2) were used to evaluate the anticancer potency of all synthesized compounds. All compounds which exhibited anticancer activities were found to be more effective (lower IC_50_ values, Table 1) on HepG2 cells than MCF-7 cells. For HepG2 cells, compounds **1a**,**b**, **3a**,**b**, **4a**–**c**, and **5a**–**c** were the most effective. On the other hand, for MCF-7 cells, compounds **2a** and **5a**–**c** were not effective against MCF-7 cancer cells. Furthermore, compounds **1b**, **3a**,**b**, and **4a**,**b** were the most potent derivatives.

## Figures and Tables

**Table 1:** IC_50_ of the tested compounds **1–5** against the MCF-7 and HepG-2 cell lines.

Compound	IC_50_ (µM)	
	MCF-7	HepG-2
**1a**	15.329 ± 0.672	6.615 ± 0.353
**1b**	10.904 ± 0.534	8.724 ± 0.474
**1c**	34.894 ± 1.273	17.011 ± 0.965
**2a**	NA	17.513 ± 0.876
**2b**	20.697 ± 0.974	18.681 ± 0.988
**2c**	24.943 ± 1.082	19.318 ± 1.057
**3a**	10.316 ± 0.546	8.063 ± 0.863
**3b**	9.911 ± 0.491	9.66 ± 0.792
**3c**	22.864 ± 0.992	16.957 ± 1.161
**4a**	9.304 ± 0.536	7.31 ± 0.595
**4b**	9.188 ± 0.545	7.166 ± 0.892
**4c**	19.552 ± 1.032	9.631 ± 0.932
**5a**	NA	7.117 ± 0.790
**5b**	NA	7.088 ± 0.784
**5c**	NA	9.568 ± 1.139
**6a**	NA	NA
**6b**	NA	NA
**6c**	NA	NA
Tamoxifen	22.368 ± 2.41	29.339 ± 1.151
Cisplatin	8.897 ± 0.37	10.93 ± 0.962

IC_50_: Compound concentration required to inhibit the cell viability by 50%, NA: not obtained.
